# Recycling of the High Valence States of Heme Proteins by Cysteine Residues of Thimet-Oligopeptidase

**DOI:** 10.1371/journal.pone.0079102

**Published:** 2013-11-01

**Authors:** Juliana C. Ferreira, Marcelo Y. Icimoto, Marcelo F. Marcondes, Vitor Oliveira, Otaciro R. Nascimento, Iseli L. Nantes

**Affiliations:** 1 Departamento de Biofísica, Universidade Federal de São Paulo, São Paulo, SP, Brasil; 2 Instituto de Física de São Carlos, Universidade de São Paulo, São Carlos, SP, Brasil; 3 Centro de Ciências Naturais e Humanas, Universidade Federal do ABC, Santo Andre, SP, Brasil; Instituto de Biociencias - Universidade de São Paulo, Brazil

## Abstract

The peptidolytic enzyme THIMET-oligopeptidase (TOP) is able to act as a reducing agent in the peroxidase cycle of myoglobin (Mb) and horseradish peroxidase (HRP). The TOP-promoted recycling of the high valence states of the peroxidases to the respective resting form was accompanied by a significant decrease in the thiol content of the peptidolytic enzyme. EPR (electron paramagnetic resonance) analysis using DBNBS spin trapping revealed that TOP also prevented the formation of tryptophanyl radical in Mb challenged by H_2_O_2_. The oxidation of TOP thiol groups by peroxidases did not promote the inactivating oligomerization observed in the oxidation promoted by the enzyme aging. These findings are discussed towards a possible occurrence of these reactions in cells.

## Introduction

Thimet oligopeptidase (EC 3.4.24.15, TOP) is a mammalian zinc metalloendopeptidase with broad tissue and subcellular compartment distribution. TOP is able to play a variety of physiological roles, such as the metabolism of small peptides 17±5 amino acids in length [1-3]. The substrates of TOP include physiological and model peptides such as neurotensin, bradykinin. somatostatin, opioids, and angiotensin I, the gonadotropin-releasing hormone in the extracellular medium [2,4-8].

In the intracellular medium, TOP promotes degradation of peptides released from proteasomes, thereby limiting the extent of antigen presentation by MHC class I molecules [9,10] and degradation of the A-peptide, a component of amyloid plaques in Alzheimer's disease [11]. The regulation of TOP activity occurs at different levels and includes posttranslational modification such as phosphorylation and glutathionylation [12,13].

Unlike other thermolysin-like metallopeptidases, TOP is unusually activated by the reduction of disulfide bond-stabilized multimers rather than by their formation. Therefore, the enzyme is activated by thiol reducing agents and this modulation by olygomerization may be a physiological regulatory mechanism [14-16].

Human TOP presents 14 cysteine residues, with 6 of those located on the outside of the enzyme where they could participate in intermolecular disulfide links. In the rat ortholog, it has been proposed that surface-accessible cysteine residues 246, 248, 253, and 682 could be involved in the enzyme oligomerization [14,17]. Cysteine residues 46 and 687 in rat TOP are also thought to be involved in multimer formation with consequent inactivation of the enzyme.

Considering the high content of cysteines in TOP structure and the multiplicity of roles played by this enzyme, it is probable the participation of these reducing amino acid residues in the redox reactions of cells.

In biological systems, thiols play a crucial role in the antioxidant defense network and are mediators of multiple metabolic, signaling, and transcriptional processes [18,19]. Thiol groups, present both in proteins and in low-molecular-mass peptides, control a considerable portion of biological properties and functions such as enzyme catalysis, protein structure, chemical modifications in proteins and redox-signaling pathways [20-22].

The redox balance of cells are also related to another class of proteins: the heme proteins. The participation of the heme proteins in oxidative stress involves pro- and antioxidant actions. In this regard, the role played by heme proteins such as catalase, cytochrome c and myoglobin, both in combat and generation of free radical and excited species, is well known [23-27]. More recently, another class of heme proteins has gained interest and has been a target of studies concerning their structure and functions: neuroglobins (Ngb) and cytoglobins (Cygb). Ngb and Cygb are two phylogenetically ancient globins [28] which, in the absence of external ligands, exhibit hexacoordinate hemes with histidine residues at both proximal and distal coordination positions [29]. Ngb and Cygb are expressed at low levels in neuronal tissues and in all tissues investigated so far, respectively. Ngb has been postulated to participate in the cellular defense against hypoxia but it is more likely be a scavenger of reactive oxygen and nitrogen species that are generated following brain hypoxia. On the other hand, Cygb is upregulated upon hypoxia, and the O_2_-binding properties of this heme protein are consistent with a role in O_2_-requiring reactions, such as those catalysed by hydroxylases [30,31].

In this study, we investigated the participation of the peptidolytic enzyme TOP as a reducing agent in the peroxidase cycle of myoglobin (Mb) and horseradish peroxidase (HRP). Mb and HRP were used as models of peroxidases promoting homolytic and heterolytic cleavage of peroxidases, respectively. The use of HRP also contributes as a control to avoid the effects of hydroxyl radical. Furthermore, despite the minor sequence similarity of Ngb and Cygb to Mb, these heme proteins also exhibit the typical 3- over-3 α-helical fold that characterizes the vertebrate globins [31,32]. Thus, Mb also represents a model for studies concerning the structure and reactivity of these proteins. An intriguing feature of Ngb is its heme hexacoordination in the absence of external ligands, observed both in the ferrous and ferric forms and responsible for the low reactivity of this protein with H_2_O_2_, [33,34]. However, the presence of a hexacoordinate heme group does not totally preclude the peroxidase activity that can be enhanced in specific conditions and deserves more detailed investigations [35-38]. The results showed a link between electron transfers from TOP sulphydryl groups to high valence states of heme proteins and enzyme activation. These novel findings encourage future investigations about the possible participation of this protease in the redox processes of cells. These studies are currently under investigation in our laboratory.

## Materials and Methods

### Chemicals

Lysogeny broth cell culture medium, Isopropyl β-D-thiogalactoside, sodium phosphate, hydrogen peroxide, DTNB, catalase and equine myoglobin were obtained from Sigma Chemical (St. Louis, MO). The expression vector pGEX-4T2, affinity chromatography using a glutathione Sepharose 4B column and thrombin (1 U/mg) were obtained from Amersham Biosciences. 

### Recombinant TOP expression

Recombinant TOP was expressed in *Escherichia coli* as a glutathione S-transferase (GST) fusion protein using the expression vector pGEX-4T2. Protein purification was conducted by affinity chromatography using a glutathione Sepharose 4B column with the protein released from the GST fusion by cleavage with thrombin. The purity of the protein was analyzed using Coomassie brilliant blue staining after 12% SDS-PAGE. After confirming that TOP homogeneity was greater than 95% (data not shown), the protein was stored at -80 °C in small aliquots.

### Recycling of oxoferryl myoglobin and electronic absorption measurements

UV-visible spectra were obtained in a photodiode spectrophotometer (Multispec 1501, Shimadzu Scientific Instruments, Columbia, MD), using quartz cuvettes with 1.0- and 0.1-cm optical path and 0.5 nm slit. The experimental procedure was as the following: 10 µM hydrogen peroxide was added to a 1 µM myoglobin buffered solution at 37 °C and the spectral changes were accompanied. Ten minutes after hydrogen peroxide addition, the maximal red shift of myoglobin Soret band is observed and then we accompanied the recycling of myoglobin to the resting form in the absence or in the presence of 100 nM catalase. The effect of TOP for myoglobin recycling was analysed by adding 10 µM TOP after convertion of myoglobin to the high valence form. When catalase is present, TOP was added concomitant with this enzyme. 

### EPR measurements

The EPR measurements were carried out using an EPR Bruker system, the ELEXSYS E580 model, equipped with a helium cryostat (Oxford, UK) and temperature controller under the following conditions: central field, 240 mT, scanning field, 400 mT, number of points, 2048, modulation amplitude, 1 mT, gain, 45 dB, temperature, 4.30 K, time constant, 20.48 ms, conversion time, 81.92 ms, microwave power, 5 mW. After the addition of reactants in different media, 120 µLl of the mixture was quickly introduced into an EPR quartz tube, cooled in liquid nitrogen, and transferred to the helium cryostat assembled to the EPR cavity to obtain the spectra.

For experiments with DBNBS spin trapping horse heart, Mb was reacted with H_2_O_2_ in the presence of DBNBS, and the radical adduct was carried out using an X-band EPR Varian system, E-109 model. For spin trapping experiments, the EPR conditions were the following: microwave frequency = 9.5077 GHz, central field, 340 mT, scanning field, 16 mT, number of points, 1024; modulation amplitude, 0.05 mT, gain, 5.0x10^5^, temperature, 293 K; time constant. 0.128 s, scan time, 180 s, microwave power, 20 mW.

EPR spectra of the Mb*^.^-DBNBS adduct were simulated by the software NLSL using the following simulation parameters: the gyromagnetic 4-* and hyperfine 4-* tensors, *A* isotropic Gaussian line width (gib_0_) and parameters related to system ordering (S_0_ e S_2_) and dynamic (4* tensor). The high amount of parameters that must be minimized makes the simulation process more difficult and complex. The simulation was carried out using typical values of gyromagnetic and hyperfine tensors for nitroxides with hyperfine isotropic split value fixed as 1.36 mT. From three tentative simulations, the best fit was obtained by aligning the yR axis with the local director zd, assuming the Euler angles of the system of rotational diffusion to be β_R_=90° e γ_R_=90°. The results are presented here. The correlation time related to the rotational diffusion tensor was of an order of 10^-7^ s, consistent with the protein rotation rate.

### Reduction of TOP

Preparations of purified recombinant TOP were incubated with TCEP (1 mM), specific sulfhydryl reductants. After incubation, TOP preparations were filtered through PD-10 (GE) to completely remove the reductants. After the determination of protein concentration, aliquots of reduced TOP preparations were taken for further incubation, SDS-PAGE, hydrolytic assays, and EPR assays.

### Determination of TOP-reduced Cys residues

These assays were performed as previously described [12,39]. Briefly, 150-250 μg sulfhydryl-modified TOP was re-suspended in 300 μL of 30 mM Tris, pH 7.4, and containing 8M urea. After complete dissolution, samples were taken for reading at 280 nm to determine the concentration of TOP stock solution. Protein concentration was calculated as described below, using the extinction coefficient at 280 nm (ɛ _280_ nm) determined for TOP as being 78, 240 M^-1^.cm^-1^. Determination of thiol content of TOP in different conditions was performed by the addition of 10 μM DTNB (final concentration) to the samples and incubated for 40 min in the dark, followed by reading at 412 nm. The concentration of the Cys-TNB complex was deduced from the ɛ _412 nm_ value equal to 14,150 M^-1^.cm^-1^. The number of reduced Cys residues was calculated by the molar ratio protein/Cys-TNB complex.

The Cys content determined for TOP samples treated with Mb or HRP comes from the exclusive contribution of TOP. Except for human myoglobin, no other species of known mammalian Mb possesses a cysteine residue [40]. HRP could not contribute to the SH content of the samples in which it is present because this enzyme has 8 cysteine residues that are participating of four disulfide bonds [41]. Regarding the dosage of the SH content of TOP treated with hydrogen peroxide, it is not necessary to submit it to TOP washing. That is because an excess of DTNB is added at the end of the incubation, blocking all SH groups available at that moment. Therefore, even though some residual hydrogen peroxide could be present at that moment, its low concentration is not competitive with the DTNB content.

### Mass spectrometry

Mb (2 μg/μL) was incubated with H_2_O_2_ and DBNBS for 2 hours at 37°C. The prepared samples were directly analyzed by Maldi-ToF/MS (Bruker Daltonics Microflex LT). Aliquots (1 μL) from the incubator and the α-cyan (10 mg/mL) matrix solution were spotted (1:1) into a stainless steel Maldi-ToF target plate and dried at room temperature before analysis. Mass spectra were obtained with an instrument operating in linear positive ion mode, previously calibrated with angiotensin I and II, substance P, bombesin, ACTH, and somatostatin. For each sample, mass spectra were acquired by accumulating 50 laser shots at 32% laser power in the m/z range of 0.6-25 kDa. The conditions of the instrument were: a pulsed ion extraction delay of 260 ns, ion source voltage one, 20 kV, ion source voltage two, 18.50 kV, and ion source lens voltage 8.50 kV.

## Results and Discussion

### TOP accelerates the recycling of the high valence states of heme proteins to the resting form

**Figure 1:**
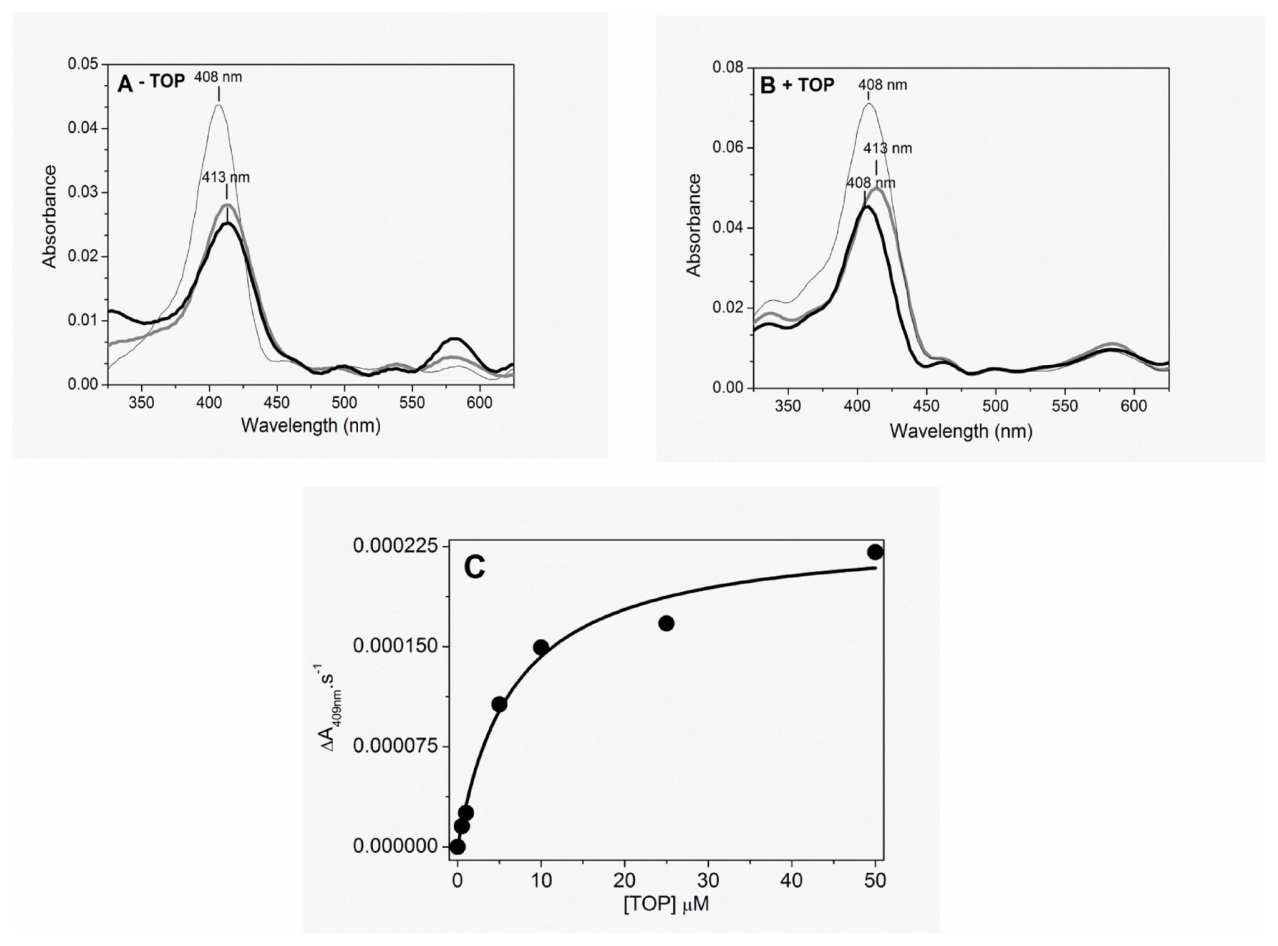
Catalytic cycle of Mb. A) Absence of TOP. Electronic absorption spectra of 0.5 µM Mb before (thin solid line),10 min (gray line) and 120 min (thick solid line) after the addition of 5 µM H_2_O_2_. B) Presence of TOP. Electronic absorption spectra of 0.5 µM Mb before (thin solid line),10 min (gray line) and 35 min (thick solid line) after the addition of 5 µM H_2_O_2_. C) Effect of TOP concentration on the rate of Mb Compound II recycling to the resting form. After normalization of the spectra of Mb Compound II and recycled Mb by the maximal intensity of Soret band, the rate of oxoferryl Mb recycling to the resting state was calculated by the difference of intensity at 408 nm and the normalized spectra of the recycled Mb. The delta normalized absorbance at 408 nm was divided by the time of recycling and plotted as a function of TOP concentration. Different TOP concentrations were added 10 min after hydrogen peroxide addition (maximal Soret band red shift). Immediately after the formation of Compound II, 5 µM of TOP was added to the medium. These results are representative of a set of two independent experiments with standard deviation of 15%. The reaction was carried out at 37 °C, in 20 mM Tris buffer pH 7.4 treated with Chelex-100®.

Figure 1 shows the changes in the Fe^3+^Mb electronic absorption spectrum promoted by H_2_O_2_ in the absence (panel A) and in the presence (panel B) of TOP. The thin black line in Figure 1A represents the spectrum of Fe^3+^Mb before the addition of H_2_O_2_ in the absence of TOP. The addition of H_2_O_2_ led to a red shift in the Soret band (408 nm to 413 nm) consistent with the formation of compound II, the oxoferryl form of heme iron, (Figure 1A, thick gray line). In this condition, the spectrum of the high valence state of Mb did not exhibit changes suggestive of the heme iron recycling to the resting state, even after a 120 min incubation with H_2_O_2_ (Figure 1A, thick black line). Recycling of Mb was observed 50 min after the conversion to high valence forms when 100 nM catalase was added (not shown). However, in the presence of TOP and in the absence of catalase, recycling of the Mb high valence state to the resting form was observed ~35 min after the addition of H_2_O_2_ (Figure 1B, thick black line). When the excess of hydrogen peroxide was removed by the addition of catalase, the Mb recycling promoted by TOP was observed 25 min after the peptidolytic enzyme addition (not shown). In both conditions, Soret band bleaching was detected, probably due to the oxidative attack of free radicals on the heme group ring. 

The rate of oxoferryl Mb recycling to the resting state exhibited a hyperbolic dependence on the concentration of 40% reduced TOP (Figure 1C). In this condition, TOP presents six external cysteine residues in the reduced form, and saturation was observed at above 10 µM TOP that corresponds to more than 60 µM SH groups. The saturation kinetics also indicates a binding affinity between Mb and TOP. The rate of oxoferryl Mb recycling to the resting state was calculated using the following procedure. The spectra of Mb Compound II and recycled Mb were normalized by the maximal intensity of Soret band. The intensity of normalized Soret band of Mb compound II at 408 nm was subtracted from the unity (intensity of Soret band peak of recycled Mb). The delta normalized absorbance at 408 nm was divided by the time of recycling and plotted as a function of TOP concentration. The different TOP concentrations were added 10 min after H_2_O_2_, when the maximal Soret band red shift was observed indicating the formation of Compound II. It is important to note that, when TOP is absent, Mb exhibits partial bleaching after recycling. It was observed that, in a concentration-dependent manner, TOP not only accelerated the Soret band blue shift back to the resting value as well as decreased the Soret band bleaching.

**Figure 2:**
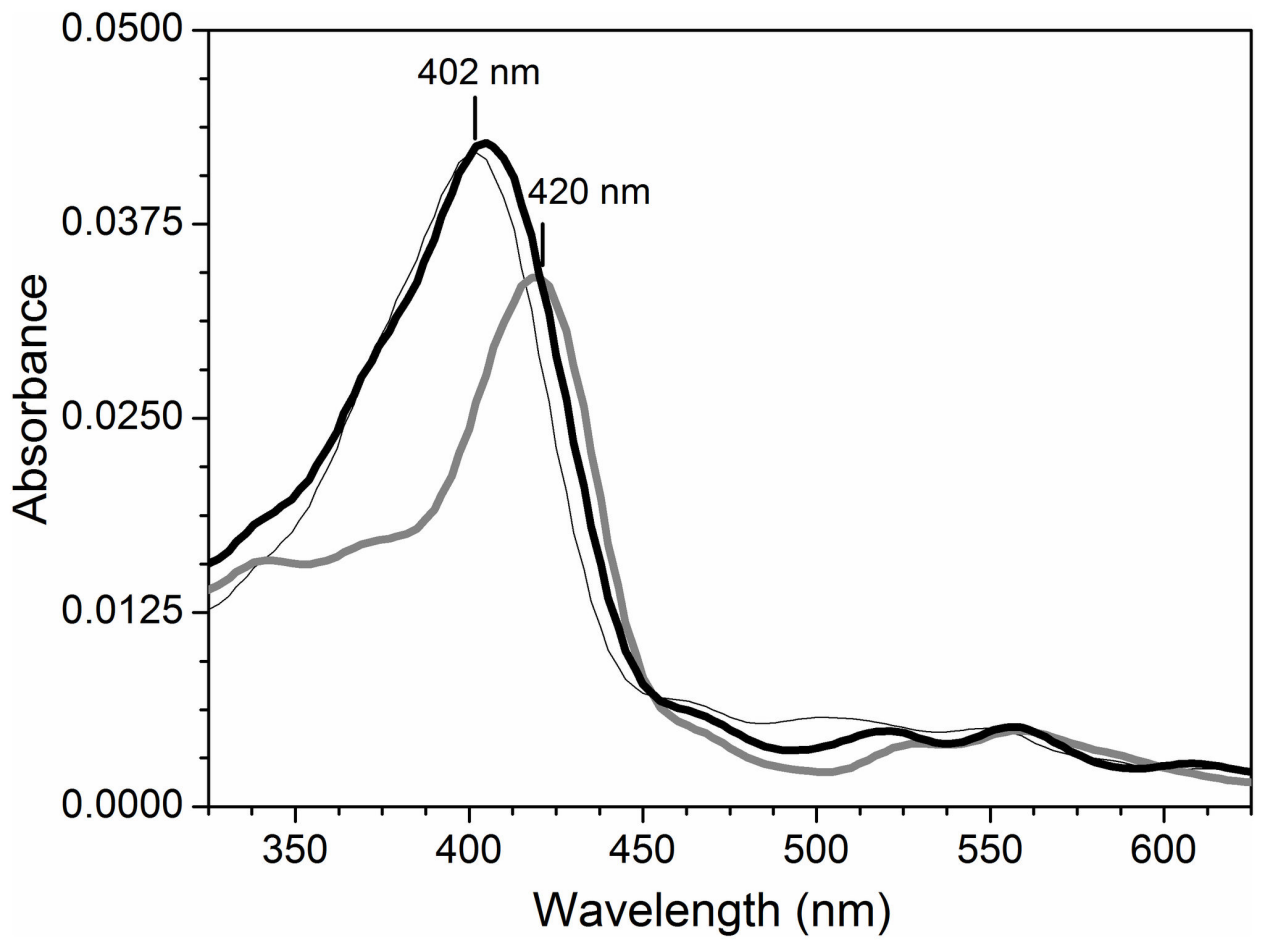
Catalytic cycle of HRP in the presence of TOP. UV-vis spectrum of 0.5 µM HRP before (thin solid line) 10 min (dotted line) and 17 min (thick solid line) after the addition of 5 µM H_2_0_2_. Immediately after the formation of Compound II, 5 µM of TOP was added to the medium. The reaction was carried out at 37 °C, in 20 mM Tris buffer pH 7.4 treated with Chelex-100 ®.

To test the capacity of TOP to recycle a typical peroxidase promoting heterolytic scission of the *0-0* bond of peroxides, the TOP was added to HRP challenged by H_2_O_2_, (Figure 2). In the absence of TOP, the recycling of HRP to the resting form required 60 min (not shown). However, in the presence of TOP, HRP was recycled to the resting form after a 17 min incubation with H_2_O_2_ (Figure 2, thick black line). For HRP, in both conditions, the recovery of the resting form occurred without bleaching the Soret band. This result is consistent with the heterolytic scission of H_2_O_2_ not associated with generation of free radicals.

**Figure 3:**
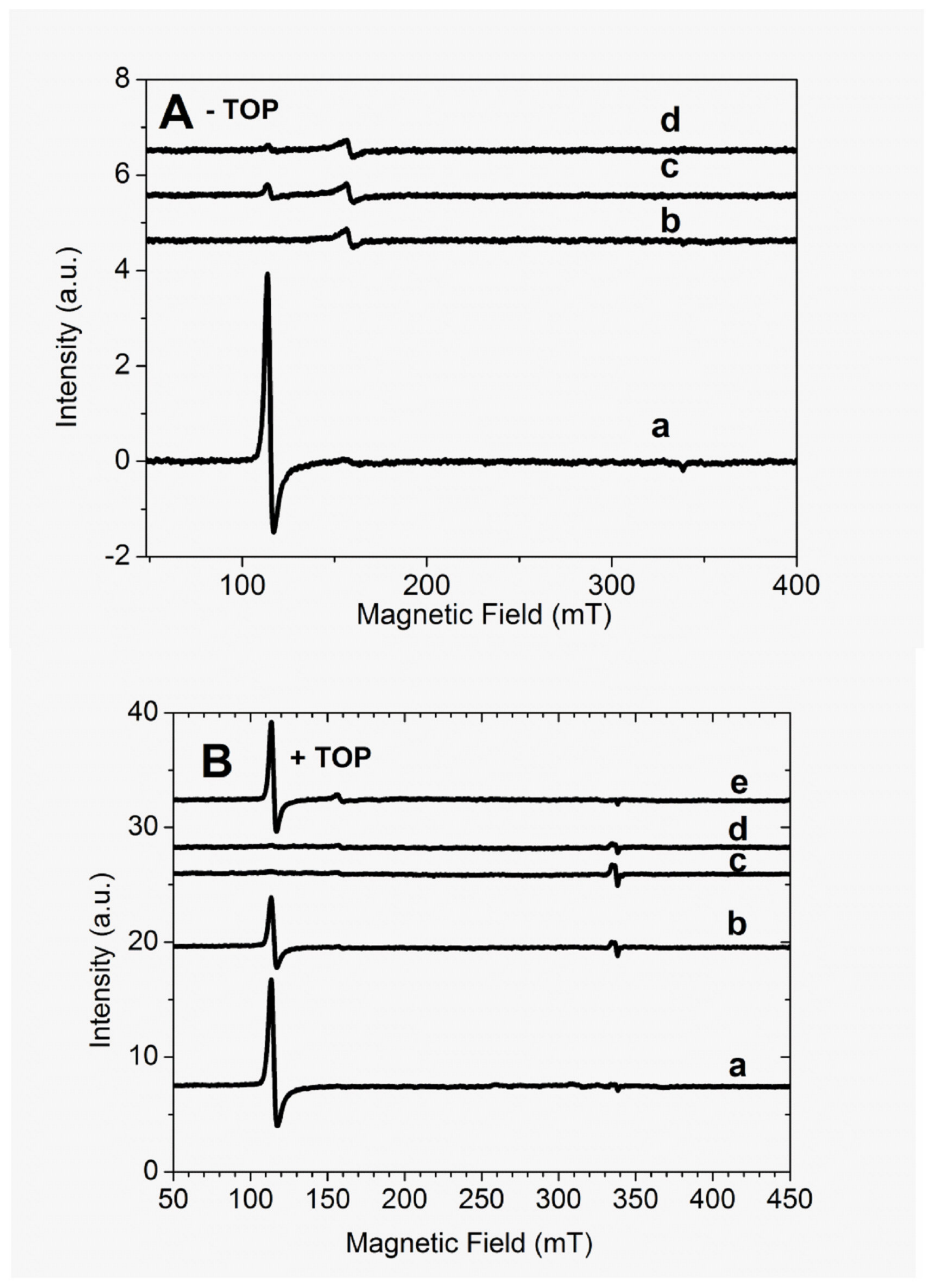
EPR spectra of Mb. A) Absence of TOP. Line a = 5 µM Fe^3+^Mb, lines b, c and d are the respective EPR spectra obtained immediately, 30 min, and 180 min after the addition of 50 µM H_2_0_2_. B) Presence of 20 µM TOP: Line a = 5 µM Fe^3+^Mb, lines b, c, d and e are the respective EPR spectra obtained immediately, 30, 60, and 90 min after the addition of 50 µM H_2_O_2_. The expanded field view shows the signal of a free radical signal overlapped on the g2 component of EPR spectrum of heme iron obtained 30 min after the addition of H_2_O_2_. When present, TOP was previously treated with 1 mM TCEP. The concentration of DMPO = 20 mM. EPR conditions were: microwave frequency = 9.47177 GHz, central field, 240 mT, scanning field, 400 mT, number of points, 2048, modulation amplitude, 1 mT, gain, 45 dB, temperature, 4.30 K, time constant, 20.5 ms, conversion time, 81.9 ms, microwave power, 5 mW. The reactions were carried out in 20 mM Tris buffer pH 7.4 treated with Chelex-100®.

To detect and characterize any radical species formed during the Mb peroxidase cycle in the absence (Figure 3A) and in the presence of TOP (Figure 3B), the reaction of Mb with H_2_O_2_ was accompanied by EPR spectroscopy at helium liquid temperature. The heme iron EPR spectra were obtained at different reaction time intervals with samples frozen at defined times and run at the indicated temperature. The EPR spectrum of native Mb obtained in 20 mM Tris buffer (line *a*), pH 7.4, reveals, at the low field region, the presence of Fe^3+^ heme iron in the high spin form (g_┴_ = 5.85 and g// = 2.00) with spin 5/2 and axial symmetry. The addition of H_2_O_2_ led to the disappearance of the Fe^3+^ EPR signal, which was in accordance to the formation of compound II, an EPR-silent species (Figure 3A) [40,42]. The g = 4.3 signal could be assigned to an oxidized form of the porphyrin ring and is normally associated with Soret band bleaching. Recycling of high valence states of Mb was not detected up to 180 min after the addition of H_2_O_2_, (Figure 3A, line *d*). In the presence of TOP, immediately after the addition of H_2_O_2_ (line *b*), a reminiscent Mb heme iron signal was observed. Significant recycling of Mb Compound II to the resting form was observed 90 min after the addition of H_2_O_2_, (Figure 3B). The recycling of the Mb high valence state by the presence of TOP led to a high yield of free radical generation.

### TOP prevented the formation of Mb tryptophanyl DBNBS adduct

**Figure 4:**
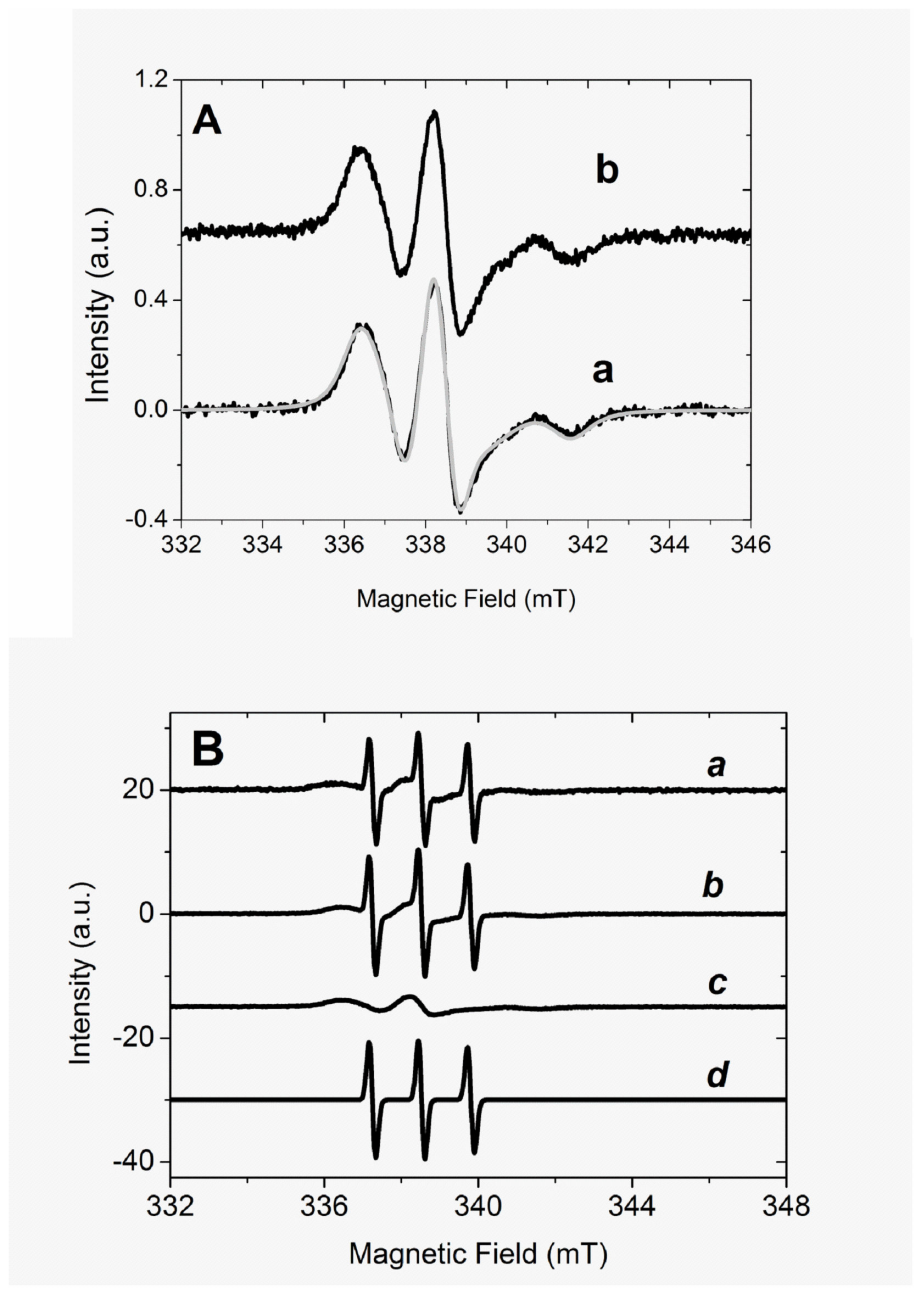
Experimental EPR spectra of tryptophanyl DBNBS adducts obtained from the reaction mixture containing Mb and H_2_O_2_. A) Absence of TOP. Line a and b correspond, respectively, to the EPR spectra of 20 mM DBNBS adduct obtained immediately and 10 min after the addition of 50 µM H_2_O_2_ to 5 µM Fe^3+^Mb solution. The light gray line, overlapped on line a, corresponds to the spectrum simulated by NLS software, B) Presence of 20 µM TOP: Lines a, b, c and d correspond to the experimental EPR spectrum, simulated composite EPR spectrum, and the simulated rigid and free rotating components, respectively. The spectrum was obtained immediately after the addition of 50 µM H_2_O_2_ in 5 µM Fe^3+^Mb solution. When present, TOP was previously treated with 1 mM TCEP. EPR conditions were: microwave frequency = 9.5077 GHz, central field, 340 mT, scanning field, 16 mT, number of points, 1024, modulation amplitude, 0.05 mT, gain, 5.0x10^5^, temperature, 293 K, time constant, 0.128 s, scan time, 300 s, microwave power, 20 mW. The reaction was performed in buffer Tris 20 mM previously treated with Chelex-100 ®, pH 7.4, at 25° C.

The scission of H_2_O_2_ by Mb converts the heme group to its oxo-ferryl form and an amino acid residue-centered free radical [43]. Data in the literature has reported multiple sites of trapping in Mb challenged by H_2_O_2_. Tyrosine 103 was identified as the preferential site for DMPO trapping and tryptophanyl for DBNBS trapping. Detection of DMPO thiyl adduct was unsuccessful, probably because the occurrence of secondary reactions that abolish the signal. The similarity of the oxidative potentials of tyrosine and tryptophan support the possibility of unpaired electron density being spread over these and other amino acid residues in a population of Mb molecules [41,43]. Addition of H_2_O_2_ to Mb in the presence of DBNBS resulted in the detection of an EPR spectrum of Mb^•^-DBNBS consistent with that of a partially immobilized nitroxide (Figure 4A) [42,44]. No EPR signal was detected when any of the reactants was present alone (not shown). The EPR spectra of DBNBS adduct obtained in the above conditions was simulated as described in materials and methods and the result is presented as a gray line overlapped on line *a*. The simulation resulted in a high value of Gaussian line width (0.377 mT) that can be attributed to a high heterogeneity of the microenvironment around the nitroxide radical. This proposal is consistent with the structure proposed by Gunther et al. [43,45] for the tryptophanyl DBNBS adduct, since hydrogen and brome atoms (I_H_=1/2 e I_Br_=3/2) are neighbors of the NO moiety. In this condition, the super hyperfine interactions not resolved by CW EPR are responsible for the increase of additional line width. The lack of additional hyperfine structure is consistent with a tertiary carbon- centered radical adduct having no bonds to atoms with nuclear spin such as nitrogen. Such structural features are satisfied by the C-3-centered radical of the tryptophan indol ring.

Surprisingly, in the presence of TOP, the EPR spectrum bearing the features of a partially immobilized tryptophanyl nitroxide radical was not detected. The EPR spectrum detected in the presence of TOP exhibited the spectral feature of free rotating adduct (Figure 4B, EPR spectrum *a*). The replacement of EPR spectra of partially immobilized adducts by another, bearing the features of a free rotating adduct, was previously obtained after digestion of MB^*^-DBNBS adduct by pronase [42, 45]. However, TOP is specific for oligopeptides and could not promote Mb digestion. The simulation of the EPR spectrum of DBNBS adducts obtained in the presence of TOP (Fig, 4B. EPR spectrum *b*) revealed a composite spectrum with the contribution of both rigid (Figure 4B, EPR spectrum *c*) and free rotating (Figure 4B, EPR spectrum *d*) adducts. The free rotating adduct EPR signal exhibited EPR parameters a_N_ = 1.280 mT, *g* = 2.0066, with line width a = 0.170 mT. h = - 0.04 and c = 0.05 (ΔH = a+b*m_i_ + c*m_1_^2^) [44,46]. This result suggests that the reducing protein TOP prevented the formation of a globin radical. Another possibility to be considered is the binding of TOP to Mb structure impairing the access of DBNBS to the globin radical. Whatever factor prevents the formation of DBNBS adduct, the free spin trapping became available as a target for a peroxidase oxidation [46]. 

**Figure 5:**
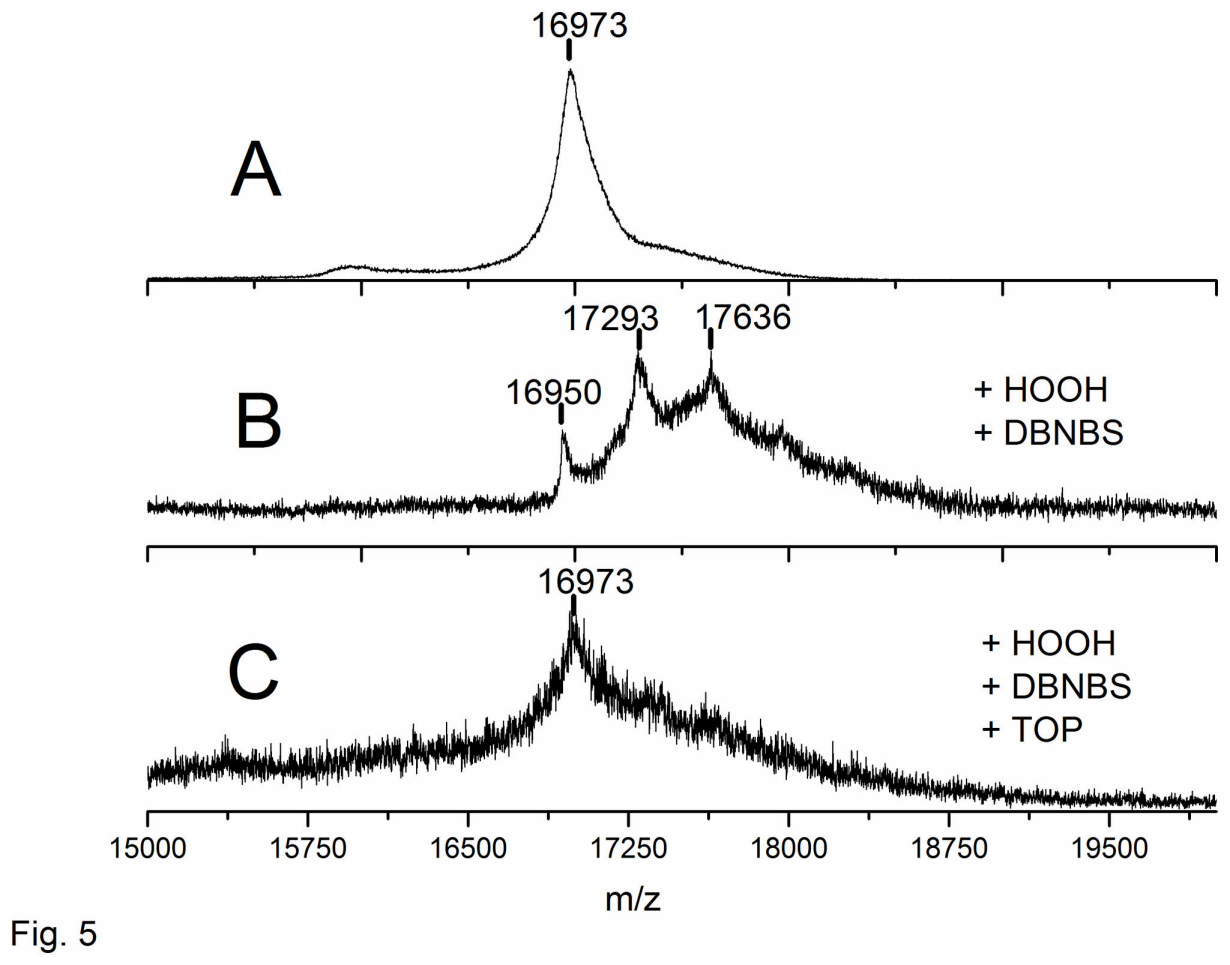
Maldi-ToF mass spectra of Mb incubated at different conditions: absence of H_2_O_2_. (A), the products of a 10 min reaction with H_2_O_2_, in the presence of 10 mM DBNBS obtained in the absence (B) and in the presence (C) of TOP. Reactions were carried out in 20 mM tris buffer pH 7.4.

The EPR analysis was complemented by the Maldi-ToF technique. Maldi-ToF analysis of Mb revealed a mass = 16973 Da consistent with Mb plus a sodium ion (Figure 5A). In the presence of TOP, the Mb Maldi-ToF spectrum revealed additional peaks at larger mass values (not shown). The values found in this condition - 17168 Da and 17298 Da — suggest, respectively, the donation of 3 and 5 Zn^2+^ ions from TOP to Mb structure and thus a significantly strong interaction between the protein structures. The donation of Zn^2+^ from TOP to Mb is not surprising because the affinity of Mb to Zn^2+^ is well known [45,46,47,48]. This finding is consistent with the kinetic data (Figure 1C) and reinforces the possibility that TOP binding to Mb structure prevents the access of DBNBS to the globin free radical. 

Analysis of the Mb-DBNBS samples by Maldi-ToF demonstrated the formation of a myoglobin-derived product with a mass increase of 343 Da (Figure 5B). These data are consistent with the addition of DBNBS minus 2 protons, as expected for the formation of a covalent adduct of DBNBS in myoglobin structure (Figure 5B). This ion was not detected in the control samples (not shown). The increase of 343 Da was not obtained in the presence of TOP (Figure 5C) indicating that the Mb tryptophanyl DBNBS adduct was not formed.Recycling of Mb high valence states occurred at expenses of the oxidation of TOP thiol groups 

**Figure 6:**
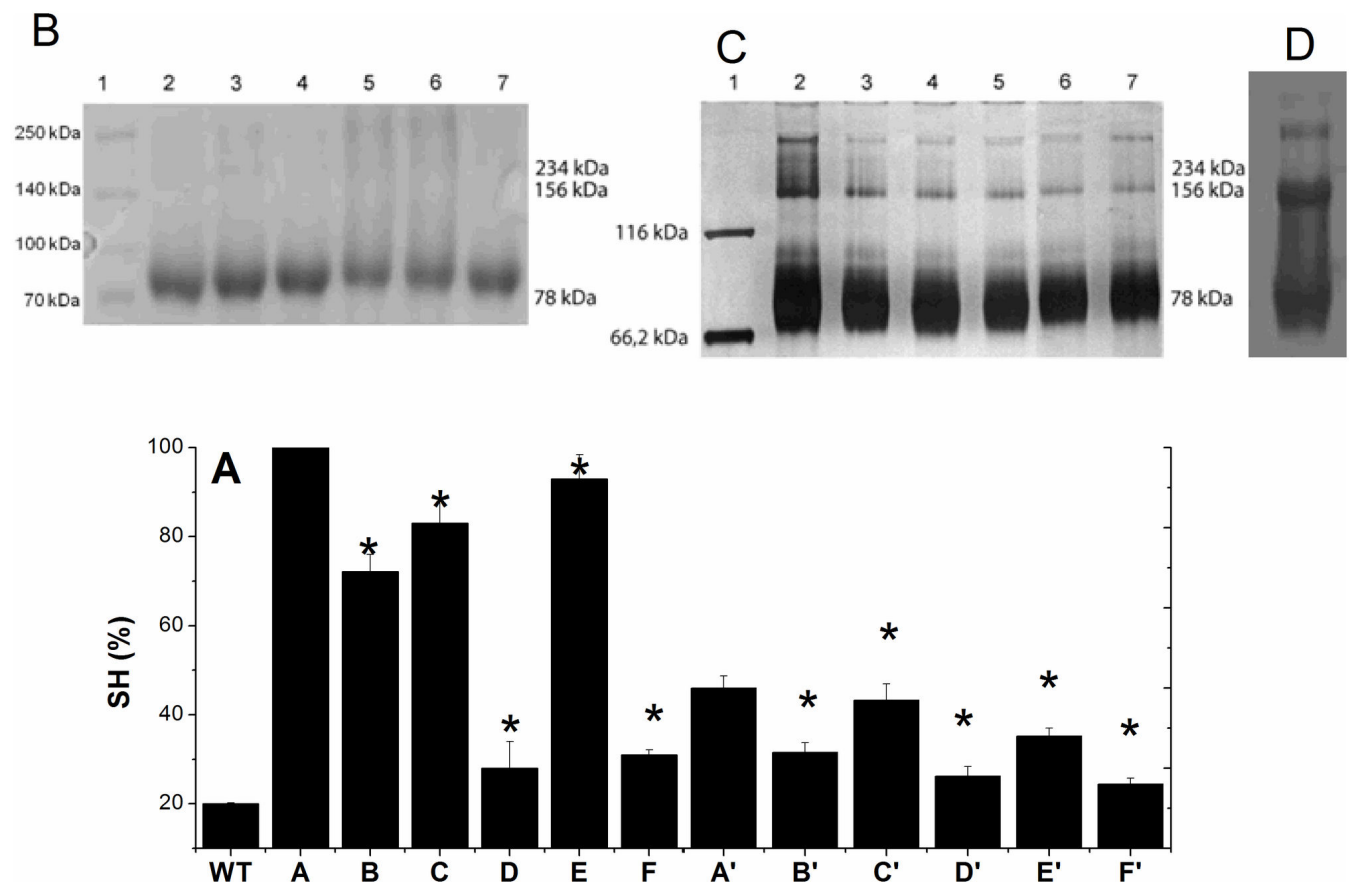
SH content and aggregates of TOP treated with 10 mM (100% reduced cysteines) and 1 mM (40% reduced cysteines) TCEP. A) WT) wild type TOP, A) + 10 mM TCEP, B) + 10 mM TCEP and 5 µM H_2_O_2_, C) + 10 mM TCEP and 0.5 µM Mb, D) + 10 mM TCEP and 0.5 µM Mb and 5 µM H_2_O_2_, E) + 10 mM TCEP and 0.5 µM HRP, F) + 10 mM TCEP and 0.5 µM HRP and 5 µM H_2_O_2_, A') + 1 mM TCEP, B') + 1 mM TCEP and 5 µM H_2_O_2_, C') + 1 mM TCEP and 0.5 µM Mb, D') + 1 mM TCEP and 0.5 µM Mb and 5 µM H_2_O_2_, E') + 1 mM TCEP and 0.5 µM HRP, F') + 1 mM TCEP and 0.5 µM HRP and 5 µM H_2_O_2_. B) SDS-PAGE of TOP at the following conditions: lanes 1 to 7 correspond, respectively, to molecular weight standard, 100% reduced TOP, + H_2_O_2_, + Mb, + H_2_O_2_, and Mb, + HRP, + HRP and H_2_O_2_. C) SDS-PAGE of 40%-reduced TOP at the following conditions: lanes 1 to 7 correspond, respectively, to molecular weight standard, 40% reduced TOP, + H_2_O_2_, + Mb, + H_2_O_2_, and Mb, + HRP, + HRP and H_2_O_2_. D) This panel shows the large amount of aggregates in aged TOP. The reaction mixtures were incubated for 2 hours in buffer Tris 20 mM Chelex-100 ®, pH 7.4, at 37° and contained 5 µM TOP. The SDS-PAGE of 100% reduced TOP was done with 10 μM TOP.

The recycling of the high valence states of the hemeproteins by TOP was investigated by using 40% and 100% reduced TOP. We have previously observed the loss of TOP αhelix content and activity after treatment with high concentrations of TCEP (Figure S1 A and B in File S1). Thus, the use of 40% and 100% reduced TOP was useful to determine whether the recycling of high valence states of hemeproteins is dependent of TOP structure. The favoring of Mb and HRP recycling by 40% and 100% reduced TOP, a rich sulphydryl protein, suggested the electron transfer from the lateral chain of cysteine residues to the high valence states of the heme proteins. Figure 6 shows the effect of the pro-oxidant activity of H_2_O_2_ and H_2_O_2_-induced high valence states of Mb and HRP on the number of reduced cysteine residues in the TOP structure and oligomerization. The treatment of TOP with both H_2_O_2_ and high valence states of heme proteins decreased the protease thiol content. However, the high valence states of Mb and HRP were more efficient than H_2_O_2_ in decreasing the thiol content of TOP. When 100% reduced TOP underwent pro-oxidant treatments, the decrease of thiol content was 28%, 8%, 17% and 7% for H_2_O_2_, resting Mb and resting HRP, respectively (Figure 6A, columns B, C and E). Conversely, after the treatment of TOP with the high valence states of Mb and HRP, the decrease in the TOP thiol content were 72% and 69%, respectively (Figure 6A columns D and F). The TOP sample with 40% reduced thiol content presented a decrease of 40%, 8% and 30%, after the treatment with H_2_O_2_, resting Mb and resting HRP, respectively (Figure 6A, columns B’, C’ and E’). In this condition, the high valence states of Mb and HRP also oxidized TOP more efficiently. After incubation with high valence states of Mb and HRP, the TOP exhibited a 55% and 60% decrease of the reduced thiol content (Figure 6A, columns D’ and F’). These results demonstrate that the high valence states of hemeproteins efficiently oxidized both the unfolded and native structured TOP. Consistently, unfolded 100% reduced TOP underwent more intense oxidation than the native structured 40% reduced TOP.

### TOP oxidation by H_2_O_2_ and high valence states of hemeproteins does not lead to enzyme oligomerization

Unexpectedly, the different prooxidant treatments did not promote a significant difference in the amount of TOP monomer (78 kDa) and dimer (156 kDa) as attested by SDS PAGE (Figure 6, panels B and C). The effect of H_2_O_2_ and high valence states of hemeproteins on TOP is significantly different from the effect of the aging that increases the amount of protein aggregates (Figure 6, panel D). The electrophoresis gel presented in panel B shows that oligomers are previously absent in samples containing 100% reduced TOP. Treatment of 100% reduced TOP with the pro-oxidants did not promote the formation of a significant amount of aggregated forms. Panel C clearly shows that the pro-oxidant treatment of 40% reduced TOP with H_2_O_2_ and with the resting and the high valence states of heme proteins was also unable to promote significant change in the amount of aggregate forms previously present in the sample.

**Figure 7:**
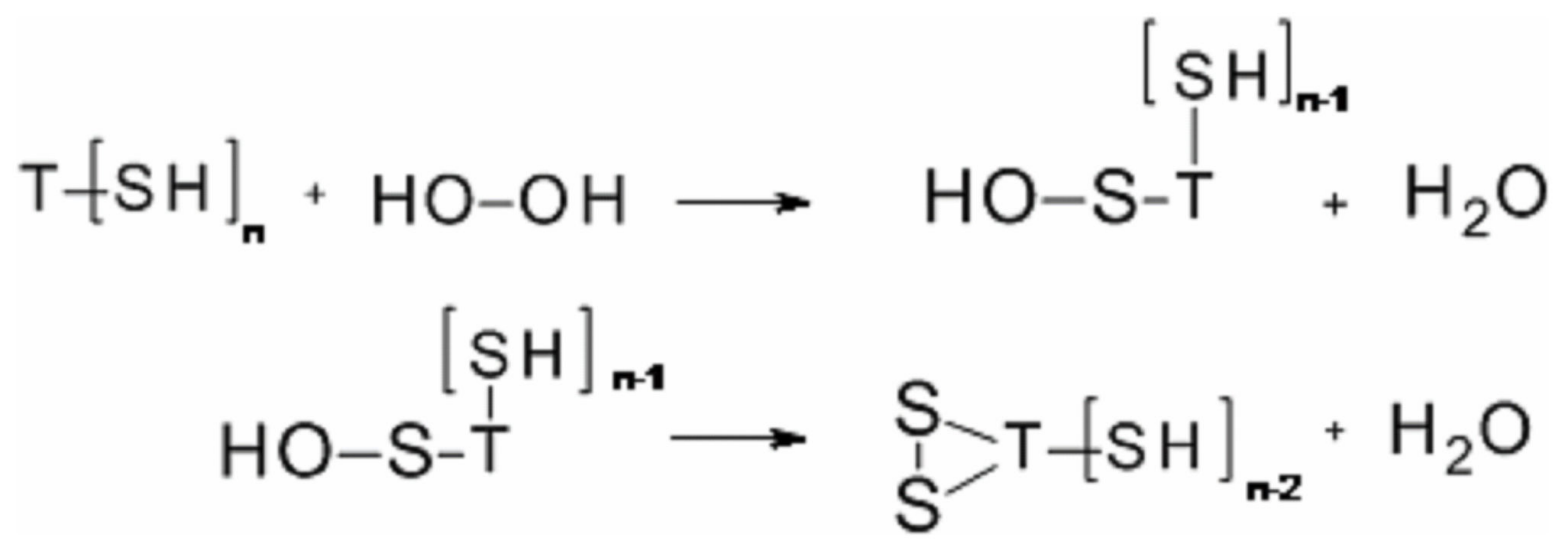
Mechanism of TOP oxidation by hydrogen peroxide.

**Figure 8:**
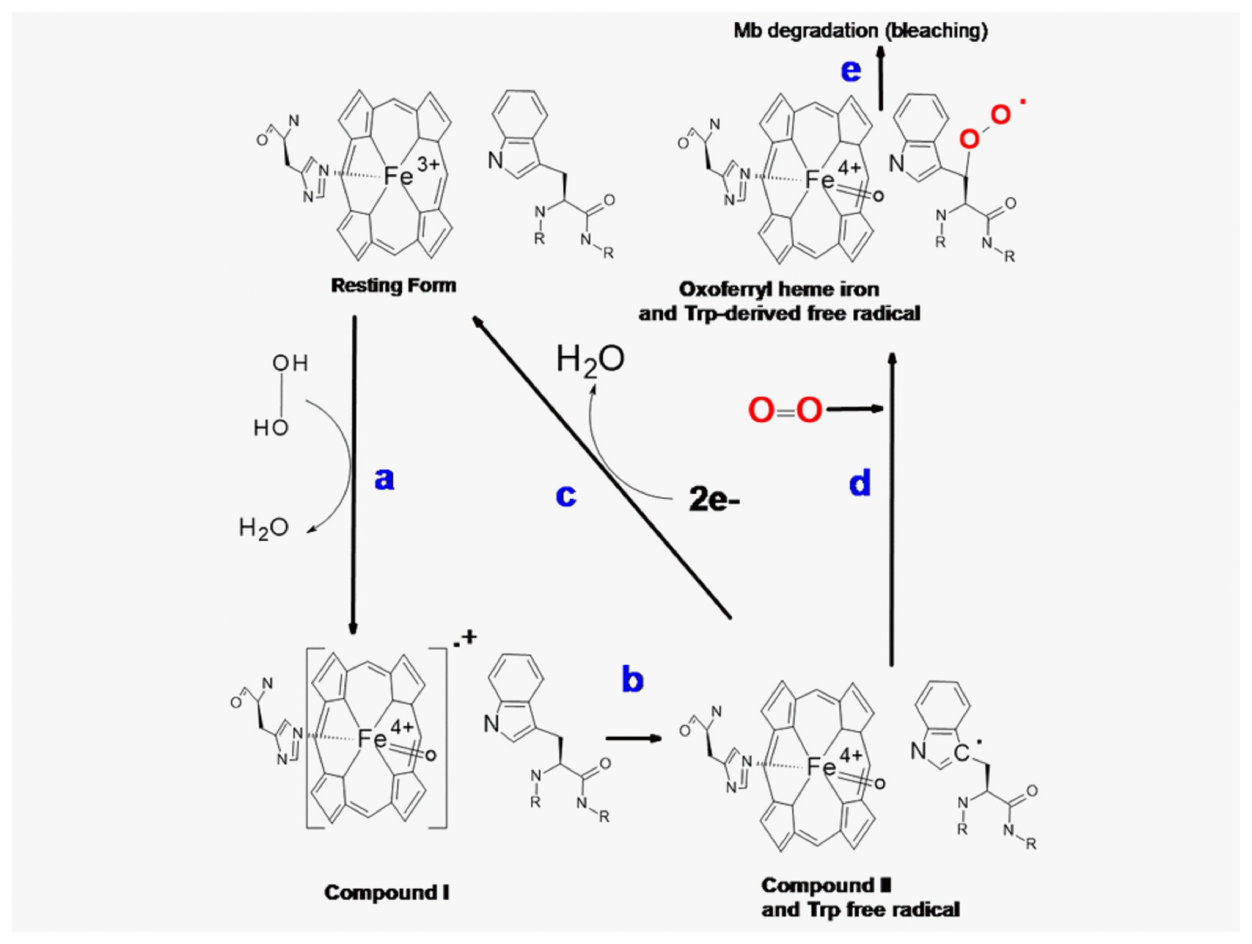
Redox cycle of myoglobin. Ferric Mb reacts with hydrogen peroxide to give the long-lived oxoferryl species and a transient protein radical (tryptophanyl radical), directly formed or resulting from electron transfer from the protein to the porphyrin π cation radical (steps *a* and *b*). The long-lived oxoferryl Mb can recycle to the resting form by two electron reduction (step c). Tryptophanyl free radical could react with molecular oxygen to form a peroxyl-derived free radical (step *d*). The protein radical may lead to the Mb degradation (bleaching) as shown in step *e*.

At this point it is important to consider the mechanisms involved in the oxidation of TOP by H_2_O_2_ and by the high valence states of hemeproteins. The mechanism of TOP thiol oxidation by H_2_O_2_ is probably the same as previously described for other proteins like bovine serum albumin [49,50]. In the uncatalysed oxidation, the mechanism probably occurs via a second order nucleophile displacement on oxygen. This mechanism may lead to thiol oxidation to sulfenic (–SOH, Figure 7), sulfinic (–SO_2_H) and sulfonic (–SO_3_H) acids and even to a combination of these thiol oxidized forms. Intramolecular disulfide has not been detected in partially oxidized TOP [12] and the formation of disulfide bonds is not expected when this enzyme is oxidized by H_2_O_2_.. However, the formation of disulfide by the spontaneous conversion of sulphenic acids to disulphides could not be discarded, and it remains to be determined (Figure 7). Considering that significant changes in the aggregated forms of TOP were not observed after treatment with hydrogen peroxide, one could conclude that the formation of intermolecular disulfide dimers were not favored. This result reinforces the idea TOP thiol groups are probably being oxidized beyond the disulfide. In cells, TOP-SOH could react with GSH to form TOP-SSG, which in turn could react with another cysteine residue to form disulfide bonds and GSH [12].Regarding the catalyzed mechanism, the proposal is depicted in Figures 8 (absence of TOP), 9 (presence of TOP) and 10 (fate of TOP thyil radical).

Figure 8 (steps a and *b*) shows that in the absence of TOP, ferric Mb reacts with hydrogen peroxide to produce the long-lived oxoferryl species and a transient protein radical (tyrosyl and tryptophanyl radicals). The protein radical may be directly formed or result from an electron transfer from the protein to the porphyrin π cation radical that has not been detected. The latter mechanism is similar to that proposed for cytochrome c peroxidase [51,52] . Because histidine 64 of Mb is not an efficient acid-base catalyst, it has been proposed that wild-type Mb may also cleave homolytically the O–O bond of the heme iron-bound peroxide, to yield Mb-II and a hydroxy radical [53]. Figure 8 only considered the heterolytic cleavage mechanism for clarity. The long-lived oxoferryl Mb can recycle to the resting form (step c) by using other Mb molecules or reminiscent H_2_O_2_ as reducing agent. Tryptophanyl free radicals could react with molecular oxygen to form a peroxyl-derived free radical (step d). The decay of protein and hydroxyl radicals might contribute for the Mb degradation (bleaching, step *e*). 

**Figure 9:**
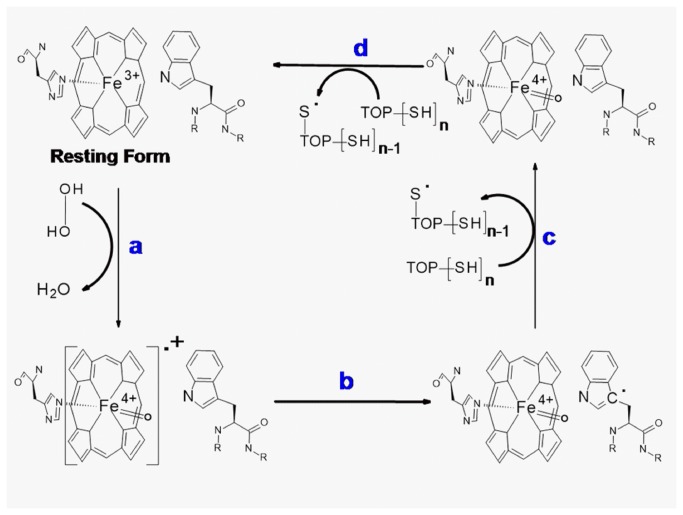
Proposed mechanism of TOP participation in the redox cycling of myoglobin. In the presence of TOP, steps *a* and *b* also occurs and the Mb “Compound I” is recycled to the resting form by using TOP thiol groups as a reducing agent (steps *c* and *d*).

**Figure 10:**
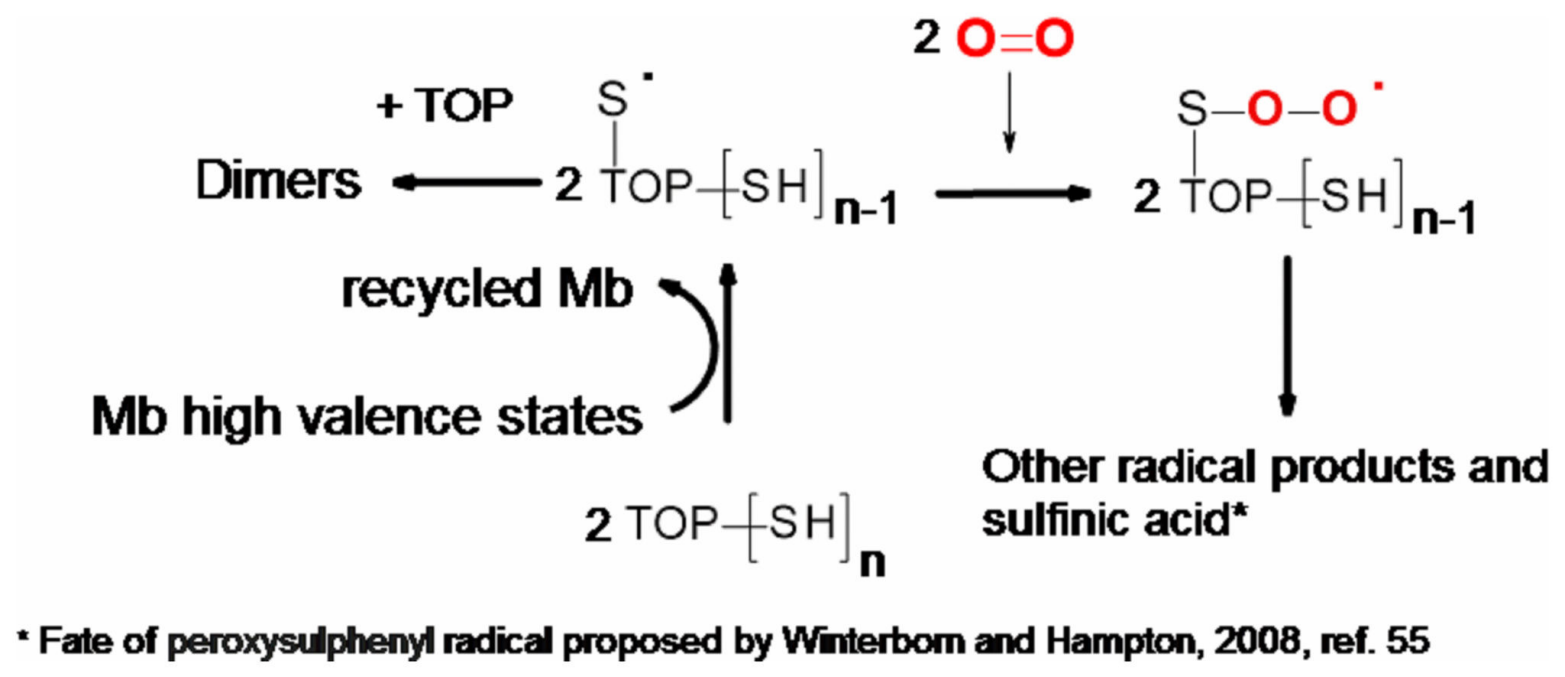
Production of TOP thiyl radical by Mb. The Mb-catalized one-electron oxidation of TOP is expected to generate thiyl free radical and the fate of this free radical is proposed according to reference 55.

In the presence of TOP (Figure 9), steps *a* and *b* also occurs, but the Mb “Compound I” is recycled to the resting form by using TOP thiol groups as a reducing agent (steps *c* and *d*). The percentage of “Compound I” recycled to the resting form by two one-electron oxidation steps (*c* and *d*) is dependent on TOP concentration. The Mb-catalized one-electron oxidation of TOP is expected to generate thiyl free radical and the fate of this free radical is proposed in Figure 10. The electrophoresis gel obtained after oxidation of 40% and 100% reduced TOP showed that oxidative oligomerization is not favored in this condition. Therefore, thiyl radicals may react with molecular oxygen generating sulfinic acid and other radical products. 

The oxidation of TOP thiol content without oligomerization is relevant because this peptidolytic enzyme is characterized by a peculiar mechanism of regulation related to oligomerization. Under non-reducing conditions, the native and recombinant forms of the enzyme are inactive and form multimers linked by inter-chain disulfide bonds. Low concentrations (0.1 mM) of dithiothreitol convert TOP to the monomeric form accompanied by up to an 8-fold increase in the activity [14]. Considering that competitive inhibitor binding occurs only in the monomeric form, it is postulated that catalytic site access is restricted in the multimeric forms [14]. Interestingly, the glutathione thiol group is also able to promote the recycling of myoglobin [54] suggesting that the use of thiol groups as reducing agents for peroxidases might participate of biological processes. The oxidation of TOP by uncatalyzed and catalyzed mechanisms might have different consequences for cells. The oxidation of TOP by hydrogen peroxide may potentially generate sulfenic acid derivatives that could be the target for glutathionilization and repair [12,55,56]. On the other hand, the catalyzed mechanism might yield peroxysulphenyl radical and consequently sulfinic acid derivatives that are irreversible products. The correlation of the TOP redox state with mechanisms of oxidation and activity will be the next step for investigation in our laboratory.

## Conclusion

According to the present study, the thiol-rich enzyme TOP is able to interact with the structure of Mb and HRP and act as a reducing agent for the H_2_O_2_-promoted oxidized forms of these heme proteins (heme iron high valence states and tyrosine and tryptophan derivative free radicals) at the expense of its thiol content. When Mb was used as a peroxidase, the presence of TOP could reduce the high valence states of MB as well as compete with oxygen for the reaction with the tryptophanyl free radical allowing for the recycling of the enzyme to the resting state (Figure 9). 

Interestingly, differentiated structural effects were observed for oxidation promoted by aging — probably slow oxidation promoted by molecular oxygen — and by the biological pro-oxidants, H_2_O_2_ and hemeproteins in high valence states. The oxidation of TOP by these biological species did not favor TOP oligomerization and consequently did not preclude the enzyme activity. Thus, the results obtained with these model systems encourages investigations about the reactions between TOP and the oxidized states of other heme proteins such as Ngb and Cygb as well as the effect of TOP on nitrosative stress related to the trapping of NO^•^ by heme proteins.

## Supporting Information

Figure S1 **Effect of different concentrations of TCEP on the percentage of thiol content of TOP and protein structure.** A – Percentage of reduced cysteine residues of 10 μM TOP treated with different concentrations of TCEP at two pH values. B – Circular dichroism (CD) spectra of TOP reduced by TCEP. The gray line represents the CD spectrum of catalytically active TOP reduced by 1 mM TCEP and the black line represents the CD spectrum inactive TOP treated with 10 mM TCEP.
(DOCX)

## References

[1] Barrett AJ, Brown MA, Dando PM, Knight CG, McKie N et al. (1995) Thimet oligopeptidase and oligopeptidase M or neurolysin. Methods Enzymol 248: 529-556. PubMed: 7674943. 7674943 10.1016/0076-6879(95)48034-x

[2] Camargo AC, Gomes MD, Reichl AP, Ferro ES, Jacchieri S et al. (1997) Structural features that make oligopeptides susceptible substrates for hydrolysis by recombinant thimet oligopeptidase. Biochem J 324: 517-522. PubMed: 9182712. 9182712 10.1042/bj3240517 PMC1218460

[3] Oliveira V, Campos M, Melo RL, Ferro ES, Camargo AC et al. (2001) Substrate specificity characterization of recombinant metallo oligopeptidases thimet oligopeptidase and neurolysin. Biochemistry 40: 4417-4425. PubMed: 11284698. 11284698 10.1021/bi002715k

[4] Orlowski M, Michaud C, Chu TG (1983) A soluble metalloendopeptidase from rat brain. Purification of the enzyme and determination of specificity with synthetic and natural peptides. Eur J Biochem 135: 81-88. PubMed: 6349998, . 6349998 10.1111/j.1432-1033.1983.tb07620.x

[5] Camargo AC, Oliveira EB, Toffoletto O, Metters KM, Rossier J (1987) Brain endooligopeptidase A, a putative enkephalin converting enzyme. J Neurochem 48: 1258- 1263. PubMed: 2880931. 2880931 10.1111/j.1471-4159.1987.tb05655.x

[6] Dahms P, Mentlein R (1992) Purification of the main somatostatin-degrading proteases from rat and pig brains, their action on other neuropeptides, and their identification as endopeptidases 24.15 and 24.16. Fun J Biochem 208: 145-154. PubMed: 1355047. 10.1111/j.1432-1033.1992.tb17168.x 1355047

[7] Lew RA, Hey NJ, Tetaz TI, Glucksman ML, Roberts JL et al. (1995) Substrate specificity differences between recombinant rat testes endopeptidase EC 3.4.24.15 and the native brain enzyme. Biochem Biophys Res Commun 209: 788-795. PubMed: 7733970. 7733970 10.1006/bbrc.1995.1569

[8] Vincent B, Jiracek J, Noble F, Loog M, Roques B et al. (1997) Contribution of endopeptidase. p. 3.4.24.15 to central neurotensin inactivation. *Fun**J**Pharmacol* 334: 49-53 10.1016/s0014-2999(97)01209-0 9346327

[9] Silva CL, Portaro FC, Bonato VL, de Camargo AC, Ferro ES (1999) Thimet oligopeptidase (EC 3.4.24.15), a novel protein on the route of MHC class I antigen presentation. Biochem Biophys Res Commun 255: 591-595. PubMed: 10049755. 10049755 10.1006/bbrc.1999.0250

[10] Saric T, Beninga J, Graef CI, Akopian TN, Rock KL et al. (2001) Major histocompatibility complex class I-presented antigenic peptides are degraded in cytosolic extracts primarily by thimet oligopeptidase. J Biol Chem 276: 36474-36481. PubMed: 11479311. 11479311 10.1074/jbc.M105517200

[11] Yamin R, Malgeri EG, Sloane JA, McGraw WT, Abraham CR (1999) Metalloendopeptidase EC 3.4.24.15 is necessary for Alzheimer's amyloid-beta peptide degradation. J Biol Chem 274: 18777-18784. PubMed: 10373494. 10373494 10.1074/jbc.274.26.18777

[12] Demasi M, Piassa Filho GM, Castro LM, Ferreira JC, Rioli. V - et al. (2008) Oligomerization of the cysteinyl-rich oligopeptidase EP24.15 is triggered by Sglutathionylation. *Free*; Radic Biol Med 44:: 1180-1190 10.1016/j.freeradbiomed.2007.12.012 18206667

[13] Tullai JW, Cummins PM, Pabon A, Roberts JL, Lopingco MC et al. (2000) The neuropeptide processing enzyme EC. p. 3 4.24.15 is modulated by protein kinase A phosphorylation. *J**Biol**Chem* 275:36514–36522 10.1074/jbc.M001843200 10969067

[14] Shrimpton CN, Glucksman MJ, Lew RA, Tullai JW, Margulies EH et al. (1997) Thiol activation of endopeptidase. Ecologist 3 4.24.15. A novel mechanism for the regulation of catalytic activity. *J**Biol**Chem* 272: 17395-17399 10.1074/jbc.272.28.17395 9211880

[15] Smith AI, Shrimpton CN, Norman UM, Clarke IJ, Wolfson AJ et al. (2000) Neuropeptidases regulating gonadal function. Biochem Soc Trans 28: 430-434. PubMed: 10961934. 10961934

[16] Tisljar U, Barrett AJ (1990) Thiol-dependent metallo-endopeptidase characteristics of Pz-peptidase in rat and rabbit Biochem .1267. pp. 531-533. PubMed: 2185743. 10.1042/bj2670531 PMC1131321 2185743

[17] Tisljar U, Barrett AJ (1989) Purification and characterization of Pz-peptidase from rabbit muscle. Arch Biochem Biophys 274: 138-144. PubMed: 2673041. 2673041 10.1016/0003-9861(89)90424-4

[18] Nantes IL, Rodrigues T, Caires AC, Cunha RL, Pessoto FS et al. (2011) Specific effects of reactive thiol drugs on mitochondrial bioenergetics. J Bioenerg Biomembr 43: 11-18. PubMed: 21279427. 21279427 10.1007/s10863-011-9328-9

[19] Arnesano F, Banci L, Bertini I, Martinelli M, Furukawa Y et al. (2004) The unusually stable quaternary structure of human Cu, Zn-superoxide dismutase 1 is controlled by both metal occupancy and disulfide status. J Biol Chem 279: 47998- 48003. PubMed: 15326189. 15326189 10.1074/jbc.M406021200

[20] Pessoto FS, Faria PA, Cunha RL, Comasseto JV, Rodrigues T et al. (2007) Organotellurane-promoted mitochondrial permeability transition concomitant with membrane lipid protection against oxidation. Chem Res Toxicol 20: 1453-1461. PubMed: 17896819. 17896819 10.1021/tx700092r

[21] Moran LK, Gutteridge JM, Quinlan GJ (2001) Thiols in cellular redox signalling and control. Curr Med Chem 8: 763-772. PubMed: 11375748. 11375748 10.2174/0929867013372904

[22] Zhang H, Xu Y, Joseph J, Kalyanaraman B (2005) Intramolecular electron transfer between tyrosyl radical and cysteine residue inhibits tyrosine nitration and induces thiyl radical formation in model peptides treated with myeloperoxidase, H_2_O_2_, and NO_2_-: [PR SPIN trapping studies. J Biol Chem 280: 40684-40698. PubMed: 16176930. 16176930 10.1074/jbc.M504503200

[23] Witting PK, Douglas DJ, Mauk AG (2000) Reaction of human myoglobin and H_2_O_2_. Involvement of a thiyl radical produced at cysteine 110. J Biol Chem 275: 20391-20398. PubMed: 10779502. 10779502 10.1074/jbc.M000373200

[24] Halliwell B (1990) How to characterize a biological antioxidant. Free Radic Res Commun 9: 1- 32. PubMed: 2159941. 2159941 10.3109/10715769009148569

[25] Pereverzev MO, Vygodina TV, Konstantinov AA, Skulachev VP (2003) Cytochrome c, an ideal antioxidant. Biochem Soc Trans 31: 1312-1315. PubMed: 14641051. 14641051 10.1042/bst0311312

[26] Goldsteins G, Keksa-Goldsteine Ahtoniemi T, Jaronen M, Arens E et al. (2008) Deleterious role of superoxide dismutase in the mitochondria I intermembrane space. J Biol Chem 283: 8446-8452. PubMed: 18171673. 18171673 10.1074/jbc.M706111200

[27] Mano CM, Barros MP, Faria PA, Prieto T, Dyszy FH et al. (2009) Superoxide radical protects liposome-contained cytochrome cagainst oxidative damage promoted by peroxynitrite and free radicals. Free Radic Biol Med 47: 841-849. PubMed: 19559788. 19559788 10.1016/j.freeradbiomed.2009.06.028

[28] Hardison RC (1996) A brief history of hemoglobins: plant, animal, protist, and bacteria. Proc Natl Acad Sci U_S_A 93: 5675-5679. doi:10.1073/pnas.93.12.5675. PubMed: 8650150, . 8650150 PMC39118

[29] Kundu S, Trent JT 3rd, Hargrove MS (2003) Plants, humans and hemoglobins. Trends Plant Sci 8: 387-393. doi:10.1016/S1360-1385(03)00163-8. PubMed: 12927972. 12927972

[30] Wittenberg JB, Wittenberg BA (2003) Myoglobin function reassessed. J Exp Biol 206: 2011-2020. doi:10.1242/jeb.00243. PubMed: 12756283. 12756283

[31] Pesce A, Bolognesi M, Bocedi A, Ascenzi P, Dewilde S et al. (2002) Neuroglobin and cytoglobin. Fresh blood for the vertebrate globin family. EMBO Rep 3: 1146-1151. doi:10.1093/embo-reports/kvf248. PubMed: 12475928. 12475928 PMC1308314

[32] Holm L and Sander C (1993) Structural alignment of globins, phycocyanins and colicin A. *FEBS Lett* 315: 301-306. 8422921 10.1016/0014-5793(93)81183-z

[33] Lardinois OM, Tomer KB, Mason RP, Deterding LJ (2008) Identification of protein radicals formed in the human neuroglobin-H_2_0_2_ reaction using immuno-spin trapping and mass spectrometry. Biochemistry 47: 10440-10448. doi:10.1021/bi800771k. PubMed: 18767815. 18767815 PMC2685255

[34] Herold S, Fago A, Weber RE, Dewilde S, Moens L (2004) Reactivity studies of the Fe(III) and Fe(II)NO forms of human neuroglobin reveal a potential role against oxidative stress. Biol Chem 279: 22841-22847. doi:10.1074/jbc.M313732200. PubMed: 15020597. 15020597

[35] Zucchi MR, Nascimento OR, Faljoni-Alario A, Prieto T, Nantes IL (2003) Modulation of cytochrome c spin states by lipid acyl chains: a continuous-wave electron paramagnetic resonance (CW-EPR) study of haem iron. Biochem J 370: 671- 678. doi:10.1042/BJ20021521. PubMed: 12429017. 12429017 PMC1223180

[36] Mugnol KC, Ando RA, Nagayasu RY, Faljoni-Alario A, Brochsztain S et al. (2008) Spectroscopic, structural, and functional characterization of the alternative low-spin state of horse heart cytochrome C. Biophys 94: 4066-4077. PubMed: 18227133. 10.1529/biophysj.107.116483 PMC2367172 18227133

[37] Figueiredo KM, Marcon RO, Campos IB, Nantes IL, Brochsztain S (2005) Photoinduced electron transfer between cytochrome c and a novel 1, 4, 5, 8- naphthalenetetracarboxylic diimide with amphiphilic character. J Photochem Photobiol B Biol 79: 1-9. doi:10.1016/j.jphotobiol.2004.11.011. 15792874

[38] Rodrigues T, de França LP, Kawai C, de Faria PA, Mugnol KC et al. (2007) Protective role of mitochondrial unsaturated lipids on the preservation of the apoptotic ability of cytochrome C exposed to singlet oxygen. J Biol Chem 282: 25577-25587. PubMed: 17567586. 17567586 10.1074/jbc.M700009200

[39] Getz EB, Xiao M, Chakrabarty T, Cooke R, Selvin PRA (1999) Comparison between the sulfhydryl reductants tris(2-carboxyethyl)phosphine and dithiothreitol for use in protein biochemistry. Anal Biochem 273: 73-80. doi:10.1006/abio.1999.4203. PubMed: 10452801. 10452801

[40] Witting PK, Mauk AG (2000) Reaction of Human Myoglobin and H_2_O_2_ Electron Transfer Between Tyrosine 103 Phenoxyl Radical and Cysteine 110 Yields a Protein-Thiyl Radical. J Biol Chem 275: 20391–20398. PubMed: 10779502. 11278969 10.1074/jbc.M011707200

[41] Welinder KG (1979) Amino Acid Sequence Studies of Horseradish Peroxidase Amino and Carboxyl Termini, Cyanogen Bromide and Tryptic Fragments, the Complete Sequence, and Some Structural Characteristics of Horseradish Peroxidase. Eur J Biochem 6: 483-502. 10.1111/j.1432-1033.1979.tb13061.x 38113

[42] Ollikka P, Alhonmäki K, Leppänen VM, Glumoff T, Raijola T et al. (1993) Decolorization of Azo, Triphenyl Methane, Heterocyclic, and Polymeric Dyes by Lignin Peroxidase Isoenzymes from Phanerochaete chrysosporium. Appl Environ Microbiol 59: 4010-4016. PubMed: 16349103. 16349103 10.1128/aem.59.12.4010-4016.1993 PMC195860

[43] Gunther MR, Tschirret-Guth RA, Witkowska HE, Fann YC, Barr DP et al. (1998) Site-specific spin trapping of tyrosine radicals in the oxidation of metmyoglobin by H_2_O_2_ . Biochem J 330: 1293-1299. PubMed: 9494099. 9494099 10.1042/bj3301293 PMC1219275

[44] Gunther MR, Kelman DJ, Corbett JT, Mason RP (1995) Self-peroxidation of metmyoglobin results in formation of an oxygen-reactive tryptophan-centered radical. J Biol Chem 270: 16075-16081. doi:10.1074/jbc.270.27.16075. PubMed: 7608169. 7608169

[45] Gunther MR, Tschirret-Guth RA, Lardinois OM, Ortiz de Montellano PR (2003) Tryptophan-14 is the preferred site of DBNBS spin trapping in the self-peroxidation reaction of sperm whale metmyoglobin with a single equivalent of H_2_O_2_ . Chem Res Toxicol 16: 652-660. doi:10.1021/tx0256580. PubMed: 12755595. 12755595

[46] Nazhat NB, Yang G, Allen RE, Blake DR, Jones P (1990) Does. p. 3, dibromo-4- nitrosobenzene sulphonate spin trap superoxide radicals. *Biochem**Biophys**Res**Commun* 166: 807-812 10.1016/0006-291x(90)90881-m 2154221

[47] Hunter CL, Maurus R, Mauk MR, Lee H, Raven EL et al. (2003) Introduction and characterization of a functionally linked metal ion binding site at the exposed heme edge of myoglobin. Proc Natl Acad Sci U_S A 100: 3647-3652. doi:10.1073/pnas.0636702100. PubMed: 12644706. 12644706 PMC152976

[48] Lepeshkevich SV, Dzhagarov BM (2009) Effect of zinc and cadmium ions on structure and function of myoglobin. Biochim Biophys Acta 1794: 103-109. doi:10.1016/j.bbapap.2008.09.024. PubMed: 18992855. 18992855

[49] Little C, O’Brien PJ (1969) Mechanism of Peroxide-Inactivation of the Sulphydryl Enzyme G1 yceraldehyde- 3 -Phosphate Dehydrogenase European. J Biochern 10: 533-538. doi:10.1111/j.1432-1033.1969.tb00721.x. 5348077

[50] Carballal S, Radi R, Kirk MC, Barnes S, Freeman BA et al. (2003) Sulfenic Acid Formation in Human Serum Albumin by Hydrogen Peroxide and Peroxynitrite. Biochemistry 42: 9906-9914. doi:10.1021/bi027434m. PubMed: 12924939. 12924939

[51] Matsui T, Ozaki Si, Watanabe Y (1997) On the Formation and Reactivity of Compound I of the His-64 Myoglobin Mutants. J Biol Chem 272: 32735–32738. doi:10.1074/jbc.272.52.32735. PubMed: 9407045. 9407045

[52] Miller VP, DePillisS GD, Ferrerj JC, Maukj AG (1992) Monooxygenase activity of cytochrome c peroxidase. J Biol Chem, 267: 8936-8942. PubMed: 1315745. 1315745

[53] Matsui T, Ozaki Si, Elaine Liong E, Phillips NG, Watanabe Y (1999) Effects of the Location of Distal Histidine in the Reaction of Myoglobin with Hydrogen Peroxide. J Biol Chem 274: 2838–2844. doi:10.1074/jbc.274.5.2838. PubMed: 9915818. 9915818

[54] Galaris D, Cadenas E, Hochstein P (1989) Glutathione-dependent reduction of peroxides during ferryl-and-met-myoglobin interconversion: a potential protective mechanism in muscle. Free Radic Biol Med 6: 473-478. doi:10.1016/0891-5849(89)90039-7. PubMed: 2744579. 2744579

[55] Winterbourn CC, Hampton MB (2008) Thiol chemistry and specificity in redox signaling. Free Radic Biol Med 45: 549-561. doi:10.1016/j.freeradbiomed.2008.05.004. PubMed: 18544350. 18544350

[56] Malvezzi A, Higa PM, Amaral AT-d, Silva GM, Gozzo FC et al. (2012) The Cysteine-Rich Protein Thimet Oligopeptidase as a Model of the Structural Requirements for S-glutathiolation and Oxidative Oligomerization. PLOS ONE 7(6): e39408. doi:10.1371/journal.pone.0039408. PubMed: 22761783. 22761783 PMC3382611

