# Supplementary material for: Recycling of the High Valence States of Heme Proteins by Cysteine Residues of Thimet-Oligopeptidase
Source: PLoS One. 2013 Nov 1;8(11):e79102. doi: 10.1371/journal.pone.0079102 (PMC3815109; doi:10.1371/journal.pone.0079102)
Supplement: Figure S1 — Effect of different concentrations of TCEP on the percentage of thiol content of TOP and protein structure. A – Percentage of reduced cysteine residues of 10 μM TOP treated with different concentrations of TCEP at two pH values. B – Circular dichroism (CD) spectra of TOP reduced by TCEP. The gray line represents the CD spectrum of catalytically active TOP reduced by 1 mM TCEP and the black line represents the CD spectrum inactive TOP treated with 10 mM TCEP. (DOCX) [file pone.0079102.s001.docx]

Supporting Information

The purification of recombinant wild type TOP yields a protein with 20% of the cysteine content in the reduced form. The reduction of cysteine residues of TOP by relatively low concentrations of TCEP (1-2 mM) is slightly favored at acidic pH (5.7). At 3 mM TCEP, the reduction of TOP cysteines is slightly favored at pH 7.4 and total reduction of the cysteine residues is obtained at the concentration range of 4-10 mM TCEP, in a pH independent manner (Fig. 1SA). The complete reduction of TOP thiol content implicates in loss of the enzyme structure (Fig. 1SB) while partial reduction of TOP with 1 mM TCEP does not affect the native structure.
